# On Positive Radial Solutions for a Class of Elliptic Equations

**DOI:** 10.1155/2014/507312

**Published:** 2014-02-25

**Authors:** Ying Wu, Guodong Han

**Affiliations:** ^1^College of Science, Xi'an University of Science and Technology, Xi'an, Shaanxi 710054, China; ^2^College of Mathematics and Information Science, Shaanxi Normal University, Xi'an, Shaanxi 710062, China

## Abstract

A class of elliptic boundary value problem in an exterior domain is considered under some conditions concerning the first eigenvalue of the relevant linear operator, where the variables of nonlinear term *f*(*s*, *u*) need not to be separated. Several new theorems on the existence and multiplicity of positive radial solutions are obtained by means of fixed point index theory. Our conclusions are essential improvements of the results in Lan and Webb (1998), Lee (1997), Mao and Xue (2002), Stańczy (2000), and Han and Wang (2006).

## 1. Introduction

The existence and multiplicity of positive radial solution for the following elliptic boundary value problem

(1) $$\begin{array}{c} -\Delta u=f\left(\left|x\right|,u\right)\;\text{for}\;\;\left|x\right|>1,\;x\in {\mathbb{R}}^{n},\;n⩾3,\\ u=0\;\text{for}\;\;\left|x\right|=1,\\ u⟶0\;\text{as}\;\;\left|x\right|⟶\infty , \end{array}$$

are considered in this paper, where *f* ∈ *C*((1, *∞*) × ℝ^+^, ℝ^+^) and ℝ^+^ = [0, *∞*).

In recent years, similar problems have been discussed by several authors; see [1–11] and references therein. The usual approaches include variational method [2, 6, 7, 9], topological method [4, 10, 11], and sub- and supersolution method [3, 5].

In [11], by using the norm-type cone expansion and compression theorem, Stańczy proved that problem (1) has at least one positive radial solution under the following conditions:

(B1) for any *M* > 0, there exists a function *p*_*M*_ ∈ *C*((1, *∞*), ℝ^+^) with ∫_1_^*∞*^ *s*(1 − *s* ^2−*n*^)*p*_*M*_(*s*)*ds* < *∞* such that
  (2) 0⩽f(s,u)⩽pM(s) for  any  (s,u)∈(1,+∞)×[0,M];  
(B2) there exists a set *B* ⊂ (1, *∞*) of positive measure such that
  (3) $$\begin{array}{c} \underset{u\to \infty }{\lim}\frac{f\left(s,u\right)}{u}=\infty \;\text{uniformly}\;\text{with}\;\text{respect}\;\text{to}\;s\in B; \end{array}$$
(B3) there exists a function *p* ∈ *C*((1, *∞*), ℝ^+^) with ∫_1_^*∞*^ *s*(1 − *s* ^2−*n*^)*p*(*s*)*ds* < *∞* such that
  (4) $$\begin{array}{c} \underset{u\to 0+}{\lim}\frac{f\left(s,u\right)}{p\left(s\right)u}=0\;\text{uniformly}\;\text{with}\;\text{respect}\;\text{to}\;s\in \left(1,\infty \right). \end{array}$$

In a recent paper [4], replacing the conditions listed above by the weaker ones

(5) $$\begin{array}{c} \underset{u\to \infty }{\lim\inf}\underset{s\in \left[c,d\right]}{\min}\frac{f\left(s,u\right)}{u}>\xi ,\;\;\underset{u\to 0+}{\mathrm{limsup}}\frac{f\left(s,u\right)}{p\left(s\right)u}<\eta \end{array}$$

uniformly with respect to  *s* ∈ (1, *∞*)  for suitable positive numbers  *ξ*  and  *η*, the authors proved that problem (1) still has at least one positive radial solution.

In the present paper, we continue the study in [4]. Under some conditions concerning the first eigenvalues corresponding to the relevant linear operators, we improve the above positive numbers *ξ* and *η* by using the fixed point index. Furthermore, we obtain several existence theorems on multiple positive radial solutions of (1). Our results cover both sub- and superlinear problems. It seems to be difficult to utilize the norm-type cone expansion and compression theorem to prove our results.

In the remainder of this section, we recall some facts on the fixed point index for completely continuous operators on a cone in the Banach space in order to prove our main results. Please refer to [12–14] for more details.

Let *E* be a real Banach space and *P* a cone in *E*. The following lemma is a well-known result of the fixed point index theory, which will play an important role in the proof of our main results.

**Lemma 1 (see [12–14])**

Let *Ω* be a bounded open set in *E* with *θ* ∈ *Ω*, $A:P\cap \bar{\Omega }\to P$ a completely continuous operator, where *θ* denotes the null element of *E*. Assume that *A* has no fixed point on *P*∩∂*Ω*.

(i) (Homotopy invariance) If *u* ≠ *μAu* for all *μ* ∈ [0,1] and *u* ∈ *P*∩∂*Ω*, then the fixed point index *i*(*A*, *P*∩*Ω*, *P*) = 1;
(ii) (omitting a direction) if there exists an element *ψ*_0_ ∈ *P*∖{*θ*} such that *u* ≠ *Au* + *μψ*_0_ for all *u* ∈ *P*∩∂*Ω* and *μ*⩾0, then *i*(*A*, *P*∩*Ω*, *P*) = 0;
(iii) (cone expansion) if ||*Au*||⩾||*u*|| for *u* ∈ *P*∩∂*Ω*, then *i*(*A*, *P*∩*Ω*, *P*) = 0;
(iv) (additivity) suppose *Ω*_1_ is an open subset of *Ω* with *θ* ∈ *Ω*_1_ and *u* ≠ *Au* for *u* ∈ *P*∩∂*Ω*_1_; then
  (6) $$\begin{array}{c} i\left(A,P\cap \Omega ,P\right)=i\left(A,P\cap \Omega_{1},P\right)+i\left(A,P\cap \left(\Omega \setminus \bar{\Omega }\right),P\right); \end{array}$$
(v) if *i*(*A*, *P*∩*Ω*, *P*) ≠ 0, then *A* has at least one fixed point in *P*∩*Ω*.

The paper is organized as follows. In Section 2 we change problem (1) into a singular two-point boundary value problem and then investigate the existence and multiplicity of its positive solutions. And some examples are presented in Section 2. Several theorems on existence and multiplicity of positive radial solutions of problem (1) are established in Section 3.

## 2. Positive Solutions of Singular Two-Point Boundary Value Problems

Looking for radial solutions *u*(*x*) = *z*(|*x*|) of (1), where *z* : ℝ^+^ → ℝ, one can substitute *v*(*t*) = *z*((1 − *t*)^1/(2−*n*)^) thus reducing (1) to the following singular two-point boundary value problem, which is singular at 1:

(7) $$\begin{array}{c} -v^{\prime \prime }=g\left(t,v\right),\;t\in \left(0,1\right),\\ v\left(0\right)=v\left(1\right)=0, \end{array}$$

where

(8) $$\begin{array}{c} g\left(t,v\right)=\frac{1}{{\left(n-2\right)}^{2}}{\left(1-t\right)}^{\left(2n-2)/(2-n\right)}f\left({\left(1-t\right)}^{1/(2-n)},v\right). \end{array}$$

It is well known that the solution of (7) in *C* ^2^[0,1] is equivalent to the solution of the following Hammerstein integral equation in *C*[0,1]:

(9) $$\begin{array}{c} v\left(t\right)=\overset{1}{\underset{0}{\int }}k\left(t,s\right)g\left(s,v\left(s\right)\right)ds,\;t\in \left[0,1\right], \end{array}$$

where Green's function

(10) $$\begin{array}{c} k\left(t,s\right)=\{\begin{array}{cc} s\left(1-t\right), & 0⩽s⩽t⩽1,\\ t\left(1-s\right), & 0⩽t⩽s⩽1. \end{array} \end{array}$$

Define an operator *A* : *C*[0,1] → *C*[0,1] as follows:

(11) $$\begin{array}{c} \left(Av\right)\left(t\right)=\overset{1}{\underset{0}{\int }}k\left(t,s\right)g\left(s,v\left(s\right)\right)ds,\;t\in \left[0,1\right]. \end{array}$$

Then the solution v* of (9) in *C*[0,1] is equivalent to the fixed point of *A* in *C*[0,1].

Let *E* = *C*[0,1] be our Banach space with the norm ||*v*|| = max⁡_*t*∈[0,1]_ | *v*(*t*)| for all *v* ∈ *E*, *P* = {*v* ∈ *E* : *v*(*t*)⩾0  for  *t* ∈ [0,1]}, and *Q* = {*v* ∈ *P* : min⁡_*t*∈[*a*,*b*]_ *v*(*t*)⩾min⁡{*a*, 1 − *b*}||*v*||}, where [*a*, *b*]⊂(0,1). It is easy to show that *P* and *Q* are cones in *E*. Let *Ω*_*r*_ = {*u* ∈ *E* : ||*u*|| < *r*} be the open ball of radius *r* in *E*. Define a set *H* by

(12) H={h∈C((0,1),ℝ+):h≢0, ∫01t(1−t)h(t)dt<+∞}.

For *h* ∈ *H*, define an operator *T*_*h*_ by

(13) $$\begin{array}{c} \left(T_{h}v\right)\left(t\right)=\overset{1}{\underset{0}{\int }}k\left(t,s\right)h\left(s\right)v\left(s\right)ds\;\text{for}\;\;v\in E. \end{array}$$

Then we have the following lemma.

**Lemma 2**

For any  *h* ∈ *H*,

(i) *T*_*h*_ : *E* → *E* is a completely continuous positive linear operator, and the spectral radius *r*(*T*_*h*_) ≠ 0 and *T*_*h*_ has a positive eigenfunction *φ*_1*h*_ corresponding to its first eigenvalue *λ*_1*h*_ = (*r*(*T*_*h*_))^−1^;
(ii) *T*_*h*_(*P*) ⊂ *Q*;
(iii) there exist *δ*_1_, *δ*_2_ > 0, such that
  (14) $$\begin{array}{c} \delta_{1}k\left(t,s\right)⩽\varphi_{1h}\left(s\right)⩽\delta_{2}k\left(s,s\right)\;for\;t,s\in \left[0,1\right]; \end{array}$$
(iv) define a functional *J*_*h*_ by *J*_*h*_(*v*) = ∫_0_^1^ *h*(*t*)*φ*_1*h*_(*t*)*v*(*t*)d*t* for *v* ∈ *E*; then *J*_*h*_(*T*_*h*_ *v*) = *λ*_1*h*_^−1^ *J*_*h*_(*v*) for  *v* ∈ *E*;
(v) let
  (15) $$\begin{array}{c} P_{0}=\left\{v\in P:J_{h}\left(v\right)⩾\lambda_{1h}^{-1}\delta_{1}||v||\right\}; \end{array}$$

then *P*_0_ is a cone in *E* and *T*_*h*_(*P*) ⊂ *P*_0_, where *δ*_1_ is defined by (14).

To prove Lemma 2, we need the following lemmas.

**Lemma 3 (see [15])**

Suppose that *E* is a Banach space, *T*_*n*_ : *E* → *E*  (*n* = 1,2, 3,…) are completely continuous operators, *T* : *E* → *E*, and

(16) $$\begin{array}{c} \underset{n\to +\infty }{\lim}\underset{||u||<r}{\max}||T_{n}u-Tu||=0,\;\;\forall r>0; \end{array}$$

then *T* is a completely continuous operator.

**Lemma 4 (see [16, 17])**

Suppose that  *T* : *E* → *E* is a completely continuous linear operator and  *T*(*P*)⊂(*P*). If there exist *ϕ* ∈ *E*∖(−*P*) and a constant  *c* > 0 such that *cTϕ*⩾*ϕ*, then the spectral radius  *r*(*T*) ≠ 0 and *T* has a postive eigenfunction corresponding to its first eigenvalue *λ*_1_ = (*r*(*T*))^−1^.

**Proof of Lemma 2**

(i) It follows from the definition of *H* that

(17) |(Thv)(t)|⩽∫01k(t,s)h(s)|v(s)|ds⩽||v||·∫01k(s,s)h(s)ds<+∞ for  any  v∈E.

Hence, by Lebesgue's dominated convergence theorem, it is easy to see that *T*_*h*_ : *E* → *E*. Obviously, *T*_*h*_(*P*) ⊂ *P* and *T*_*h*_ is a linear operator; namely, *T*_*h*_ is a positive linear operator. Next, we will show that *T*_*h*_ is completely continuous. For any natural number *n*  (*n*⩾2), let

(18) $$\begin{array}{c} h_{n}\left(t\right)=\{\begin{array}{cc} \inf_{t<s⩽1/n}h\left(s\right), & 0⩽t⩽\frac{1}{n},\\ h\left(t\right), & \frac{1}{n}⩽t⩽\frac{n-1}{n},\\ \inf_{\left(n-1\right)/n⩽s<t}h\left(s\right), & \frac{n-1}{n}⩽t⩽1. \end{array} \end{array}$$

Then *h*_*n*_ : [0,1]→[0, +*∞*) is continuous and *h*_*n*_(*t*) ⩽ *h*(*t*) for all *t* ∈ (0,1). Let

(19) $$\begin{array}{c} \left(T_{h_{n}}v\right)\left(t\right)=\overset{1}{\underset{0}{\int }}k\left(t,s\right)h_{n}\left(s\right)v\left(s\right)ds. \end{array}$$

It is clear that *T*_*h*_*n*__ : *E* → *E* is completely continuous. For any *r* > 0 and *v* ∈ *Ω*_*r*_, according to (18), (19), and the absolute continuity of integral, we have

(20) lim⁡n→∞||Thnv−Thv||  =lim⁡n→∞max⁡t∈[0,1]|∫01k(t,s)(hn(s)−h(s))v(s)ds|  ⩽||v||lim⁡n→∞∫01k(s,s)(h(s)−hn(s))ds  =||v||lim⁡n→∞∫e(n)k(s,s)(h(s)−hn(s))ds  ⩽||v||lim⁡n→∞∫e(n)k(s,s)h(s)ds=0,

where *e*(*n*) = [0,1/*n*]∪[(*n* − 1)/*n*, 1]. Therefore, by Lemma 3, *T*_*h*_ : *E* → *E* is a completely continuous operator.

It is obvious that there exists *t*_1_ ∈ (0,1) such that *k*(*t*_1_, *t*_1_)*h*(*t*_1_) > 0. Thus there is [*a*_1_, *b*_1_]⊂(0,1) such that *t*_1_ ∈ (*a*_1_, *b*_1_) and *k*(*t*, *s*)*h*(*s*) > 0 for all *t*, *s* ∈ [*a*_1_, *b*_1_]. Take *ζ* ∈ *P* such that *ζ*(*t*_1_) > 0 and *ζ*(*t*) = 0 for all *t* ∉ [*a*_1_, *b*_1_]. Then for *t* ∈ [*a*_1_, *b*_1_],

(21) (Thζ)(t)=∫01k(t,s)h(s)ζ(s)ds⩾∫a1b1k(t,s)h(s)ζ(s)ds>0.

So there exists a constant *c* > 0 such that *c*(*T*_*h*_ *ζ*)(*t*)⩾*ζ*(*t*) for all *t* ∈ [0,1]. From Lemma 4, we have that the spectral radius *r*(*T*_*h*_) ≠ 0 and *T*_*h*_ has a positive eigenfunction corresponding to its first eigenvalue *λ*_1*h*_ = (*r*(*T*_*h*_))^−1^.

(ii) To prove *T*_*h*_(*P*) ⊂ *Q*, we only need to show

(22) $$\begin{array}{c} \underset{t\in \left[a,b\right]}{\min}\left(T_{h}v\right)\left(t\right)⩾\min\left\{a,1-b\right\}||T_{h}v||\;\text{for}\;\;v\in P. \end{array}$$

In fact, for every *v* ∈ *P*, from *k*(*t*, *s*) ⩽ *k*(*s*, *s*) = *s*(1 − *s*) for *t*, *s* ∈ [0,1], we have

(23) (Thv)(t)=∫01k(t,s)h(s)v(s)ds⩽∫01s(1−s)h(s)v(s)ds for  t∈[0,1],

so

(24) $$\begin{array}{c} ||T_{h}v||⩽\overset{1}{\underset{0}{\int }}s\left(1-s\right)h\left(s\right)v\left(s\right)ds\;\forall v\in P. \end{array}$$

Notice that, for *t* ∈ [*a*, *b*],

(25) $$\begin{array}{c} k\left(t,s\right)=\{\begin{array}{cc} s\left(1-t\right)⩾s\left(1-b\right), & s⩽t;\\ t\left(1-s\right)⩾a\left(1-s\right), & t⩽s; \end{array} \end{array}$$

thus,

(26) $$\begin{array}{c} k\left(t,s\right)⩾\min\left\{a,1-b\right\}s\left(1-s\right)\;\text{for}\;\;\left(t,s\right)\in \left[a,b\right]\times \left[0,1\right]. \end{array}$$

It follows from (24) and (26) that, for all *v* ∈ *P*,

(27) (Thv)(t)=∫01k(t,s)h(s)v(s)ds⩾min⁡⁡{a,1−b}∫01s(1−s)h(s)v(s)ds⩾min⁡⁡{a,1−b}||Thv|| for  t∈[a,b].

So (22) holds; thus, *T*_*h*_ maps *P* into *Q*.

(iii) Since *φ*_1*h*_ is a positive eigenfunction of *T*_*h*_, we know from the maximum principle (see [18]) that *φ*_1*h*_(*t*) > 0 for all *t* ∈ (0,1). Note that *k*(0, *s*) = *k*(1, *s*) ≡ 0 for *s* ∈ [0,1]; we have *φ*_1*h*_(0) = *φ*_1*h*_(1) = 0. This implies that *φ*_1*h*_′(0) > 0 and *φ*_1*h*_′(1) < 0 (see [18]). Define a function Φ_*h*_ on [0,1] by

(28) $$\begin{array}{c} \Phi_{h}\left(s\right)=\{\begin{array}{cc} \varphi_{1\;h}^{\prime }\left(0\right), & s=0,\\ \frac{\varphi_{1\;h}\left(s\right)}{s\left(1-s\right)}, & s\in \left(0,1\right),\\ -\varphi_{1\;h}^{\prime }\left(1\right), & s=1. \end{array} \end{array}$$

Then it is easy to see that Φ_*h*_ is continuous on [0,1] and Φ_*h*_(*s*) > 0 for all *s* ∈ [0,1]. So, there exist *δ*_1_, *δ*_2_ > 0 such that *δ*_1_ ⩽ Φ_*h*_(*s*) ⩽ *δ*_2_ for all *s* ∈ [0,1]. Thus

(29) δ1k(t,s)⩽δ1s(1−s)⩽φ1h(s),⩽δ2s(1−s)=δ2k(s,s),

for all *t*, *s* ∈ [0,1].

(iv) From (14), *J*_*h*_(*v*) = ∫_0_^1^ *h*(*t*)*φ*_1*h*_(*t*)*v*(*t*)*ds* ⩽ *δ*_2_∫_0_^1^ *t*(1 − *t*)*h*(*t*)*v*(*t*)*dt* < +*∞* for all *v* ∈ *E*. So *J* : *E* → ℝ^1^ is well defined. For *v* ∈ *E*,

(30) Jh(Thv)=∫01h(t)φ1 h(t)(∫01k(t,s)h(s)v(s)ds)dt=∫01h(s)v(s)(∫01k(s,t)h(t)φ1 h(t)dt)ds=∫01h(s)v(s)(r1hφ1 h(s))ds=λ1 h−1Jh(v).

(v) It is easy to verify that *P*_0_ is a cone in *E*. It follows from (14) and (30) that

(31) Jh(Thv)=λ1 h−1∫01h(s)φ1h(s)v(s)ds⩾λ1 h−1δ1∫01h(s)k(t,s)v(s)ds=λ1 h−1δ1(Thv)(t) for  v∈P.

So *J*_*h*_(*T*_*h*_ *v*)⩾*λ*_1*h*_^−1^ *δ*_1_||*T*_*h*_ *v*|| for all *v* ∈ *P*. The proof is completed.

Denote

(32) $$\begin{array}{c} M_{1}={\left(\underset{t\in \left[a,b\right]}{\min}\overset{b}{\underset{a}{\int }}k\left(t,s\right)ds\right)}^{-1},\\ \lambda ={\left(\underset{t\in \left[0,1\right]}{\max}\overset{b}{\underset{a}{\int }}k\left(t,s\right)ds\right)}^{-1}. \end{array}$$

We list some conditions as follows which will be useful in this section.

(H_1_) *g* ∈ *C*((0,1) × ℝ^+^, ℝ^+^) and for any *M* > 0 there exists a function *h*_*M*_ ∈ *H* such that
  (33) $$\begin{array}{c} g\left(t,v\right)⩽h_{M}\left(t\right)\;\forall \left(t,v\right)\in \left(0,1\right)\times \left[0,M\right]; \end{array}$$
(H_2_) there exists a function *h* ∈ *H* such that
  (34) $$\begin{array}{c} \underset{v\to 0+}{\mathrm{limsup}}\frac{g\left(t,v\right)}{h\left(t\right)v}<\lambda_{1h}\;\text{uniformly}\;\text{w}.\text{r}.\text{t}.\;t\in \left(0,1\right); \end{array}$$
(H_3_) there exists a function *h* ∈ *H* such that
  (35) $$\begin{array}{c} \underset{v\to +\infty }{\mathrm{limsup}}\frac{g\left(t,v\right)}{h\left(t\right)v}<\lambda_{1h}\;\text{uniformly}\;\text{w}.\text{r}.\text{t}.\;t\in \left(0,1\right); \end{array}$$
(H_4_) liminf⁡_*v*→0+_⁡min⁡_*t*∈[*a*,*b*]_ *g*(*t*, *v*)/*v* > *M*_1_;
(H_5_) liminf⁡_*v*→+*∞*_min⁡_*t*∈[*a*,*b*]_ *g*(*t*, *v*)/*v* > *M*_1_;
(H_6_) there exists a number *l* > 0 such that
  (36) $$\begin{array}{c} g\left(t,v\right)>\lambda l\;\text{for}\;\left(t,v\right)\in \left[a,b\right]\times \left[\min\left\{a,1-b\right\}l,l\right]; \end{array}$$
(H_7_) there exists a function *h* ∈ *H* such that
  (37) $$\begin{array}{c} \underset{v\to 0+}{\mathrm{liminf}}\frac{g\left(t,v\right)}{h\left(t\right)v}>\lambda_{1h}\;\text{uniformly}\;\;\text{w}.\text{r}.\text{t}.\;t\in \left(0,1\right); \end{array}$$
(H_8_) there exist *h* ∈ *H* with *h*(*t*)≢0 for *t* ∈ [*a*, *b*] and *q* ∈ *C*(ℝ^+^, ℝ^+^) such that
  (38) $$\begin{array}{c} g\left(t,v\right)⩾h\left(t\right)q\left(v\right)\;\forall \left(t,v\right)\in \left(0,1\right)\times {\mathbb{R}}^{+}, \end{array}$$
  (39) $$\begin{array}{c} \underset{v\to +\infty }{\mathrm{liminf}}\frac{q\left(v\right)}{v}>\lambda_{1h}. \end{array}$$

**Lemma 5**

Assume (H_1_) holds. Then *A* : *Q* → *Q* is a completely continuous operator.

**Proof**

The proof is similar to that of Lemma 3.1 in [4], so we only sketch it. Under (H_1_), *A* is well defined and for every *v* ∈ *Q*, *Av* is nonnegative and continuous on [0,1]. Note the property of *k*(*t*, *s*); it is easy to see that *A*(*Q*) ⊂ *Q*. (H_1_) and Lebesgue's dominated convergence theorem ensure the continuity of *A*. Finally, by using Ascoli-Arzela theorem, we can prove that *A* is completely continuous.

**Lemma 6**

Assume (H_1_) holds.

- If (H_2_) is satisfied, then *i*(*A*, *Q*∩*Ω*_*r*_, *Q*) = 1 for sufficiently small positive number *r*;
- if (H_3_) is satisfied, then *i*(*A*, *Q*∩*Ω*_*R*_, *Q*) = 1 for sufficiently large positive number *R*;
- if (H_4_) is satisfied, then *i*(*A*, *Q*∩*Ω*_*r*_, *Q*) = 0 for sufficiently small positive number *r*;
- if (H_5_) is satisfied, then *i*(*A*, *Q*∩*Ω*_*R*_, *Q*) = 0 for sufficiently large positive number *R*;
- if (H_6_) is satisfied, then *i*(*A*, *Q*∩*Ω*_*l*_, *Q*) = 0;
- if (H_7_) is satisfied, then *i*(*A*, *Q*∩*Ω*_*r*_, *Q*) = 0 for sufficiently small positive number *r*;
- if (H_8_) are satisfied, then *i*(*A*, *Q*∩*Ω*_*R*_, *Q*) = 0 for sufficiently large positive number *R*.

**Proof**

(i) By (H_2_), there exists *r* > 0 such that

(40) $$\begin{array}{c} g\left(t,v\right)⩽\lambda_{1h}h\left(t\right)v\;\forall \left(t,v\right)\in \left(0,1\right)\times \left[0,r\right]. \end{array}$$

Define *S*_*h*_ *v* = *λ*_1*h*_ *T*_*h*_ *v* for *v* ∈ *E*; then *S*_*h*_ : *E* → *E* is a bounded linear operator with *S*_*h*_(*P*) ⊂ *Q* and the spectral radial *r*(*S*_*h*_) = 1. For every *v* ∈ *Q*∩∂*Ω*_*r*_, it follows from (40) that

(41) (Av)(t)=∫01k(t,s)g(s,v(s))ds⩽λ1h∫01k(t,s)h(s)v(s)ds=λ1h(Thv)(t)=(Shv)(t) for  t∈[0,1].

So

(42) $$\begin{array}{c} Av⩽S_{h}v\;\forall v\in Q\cap \partial \Omega_{r}. \end{array}$$

If there exist *v*_1_ ∈ *Q*∩∂*Ω*_*r*_ and *μ*_1_ ∈ [0,1] such that *v*_1_ = *μ*_1_ *Av*_1_, then it is easy to see that *μ*_1_ ∈ (0,1). Thus *τ*_1_ = *μ*_1_^−1^ > 1 and *τ*_1_ *v*_1_ = *Av*_1_ ⩽ S_*h*_ *v*_1_. By induction, we have *τ*_1_^*n*^ *v*_1_ ⩽ *S*_*h*_^*n*^ *v*_1_, *n* = 1,2,…. Then *τ*_1_^*n*^ *v*_1_ = *S*_*h*_^*n*^ *v*_1_ ⩽ ||*S*_*h*_||||*v*_1_|| and taking the supremum on [0,1] gives *τ*_1_^*n*^ ⩽ ||*S*_*h*_^*n*^||. By the spectral radius formula, we have

(43) $$\begin{array}{c} r\left(S_{h}\right)=\underset{n\to \infty }{\lim}\sqrt[{n}]{||S_{h}^{n}||}⩾\tau_{1}>1, \end{array}$$

which is a contradiction. According to the homotopy property invariance of fixed point index, we have *i*(*A*, *Q*∩*Ω*_*r*_, *Q*) = 1.

(ii) By (H_3_), there exist *η* > 0 and *ε*_0_ ∈ (0,1) such that

(44) $$\begin{array}{c} g\left(t,v\right)⩽\varepsilon_{0}\lambda_{1h}h\left(t\right)v\;\forall \left(t,v\right)\in \left(0,1\right)\times \left[\eta ,+\infty \right). \end{array}$$

From (H_1_), there is *h*_*η*_ ∈ *H* such that *g*(*t*, *v*) ⩽ *h*_*η*_(*t*) for all (*t*, *v*)∈(0,1)×[0, *η*]. Hence,

(45) $$\begin{array}{c} g\left(t,v\right)⩽\varepsilon_{0}\lambda_{1h}h\left(t\right)v+h_{\eta }\left(t\right)\;\forall \left(t,v\right)\in \left(0,1\right)\times \left[0,\infty \right). \end{array}$$

Define *S*_*h*_ *v* = *ε*_0_ *λ*_1*h*_ *T*_*h*_ *v* for *v* ∈ *E*; then *S*_*h*_ : *E* → *E* is a bounded linear operator and *S*_*h*_(*P*) ⊂ *Q*. Let *C*_1_ = ∫_0_^1^ *t*(1 − *t*)*h*_*η*_(*t*)*dt* < +*∞*. Set

(46) $$\begin{array}{c} W=\left\{v\in Q:v=\mu Av,\;\mu \in \left[0,1\right]\right\}. \end{array}$$

Next we prove that *W* is bounded. For any *v* ∈ *W*, from (45), we have

(47) v(t)=μ(Av)(t)⩽(Av)(t)=∫01k(t,s)g(s,v(s))ds⩽ε0λ1h∫01k(t,s)h(s)ds+∫01k(t,s)hη(s)ds⩽ε0λ1h(Thv)(t)+C1=(Shv)(t)+C1, t∈[0,1].

Thus

(48) $$\begin{array}{c} \left(\left(I-S_{h}\right)v\right)\left(t\right)⩽C_{1}\;\forall v\in W,\;t\in \left[0,1\right]. \end{array}$$

Since *λ*_1*h*_ is the first eigenvalue of *T*_*h*_ and *ε*_0_ ∈ (0,1), the first eigenvalue of *S*_*h*_, *r*(*S*_*h*_)^−1^ > 1. Therefore, the inverse operator (*I* − *S*_*h*_)^−1^ exists and

(49) $$\begin{array}{c} {\left(I-S_{h}\right)}^{-1}=I+S_{h}+S_{h}^{2}+\cdots +S_{h}^{n}+\cdots . \end{array}$$

It follows from *T*_*h*_(*P*) ⊂ *Q* that (*I* − *S*_*h*_)^−1^(*P*) ⊂ *Q*. Hence, we have from (48) that

(50) $$\begin{array}{c} v\left(t\right)⩽{\left(I-S_{h}\right)}^{-1}C_{1}\;\forall v\in W,\;t\in \left[0,1\right]. \end{array}$$

That is, *W* is bounded. Choose *R* > {*η*, sup⁡*W*}; then *v* ≠ *μ* *Av* for all *μ* ∈ [0,1] and *v* ∈ *Q*∩*Ω*_*R*_. By the homotopy invariance property of fixed point index, we have *i*(*A*, *Q*∩*Ω*_*R*_, *Q*) = 1.

(iii)–(v) have been proved in [4], so we skip it.

(vi) By (H_7_), there exists *r* > 0 such that

(51) $$\begin{array}{c} g\left(t,v\right)⩾\lambda_{1h}h\left(t\right)v\;\forall \left(t,v\right)\in \left(0,1\right)\times \left[0,r\right]. \end{array}$$

For any *v* ∈ *Q*∩*Ω*_*r*_, we have

(52) (Av)(t)⩾λ1h∫01k(t,s)h(s)v(s)ds=λ1h(Thv)(t) for  t∈[0,1].

Without loss of generality, we can suppose that *A* has no fixed point on *Q*∩∂*Ω*_*r*_. Suppose that there exist *v*_1_ ∈ *Q*∩∂*Ω*_*r*_ and *μ*_1_⩾0 such that *v*_1_ = *Av*_1_ + *μ*_1_ *φ*_1*h*_. Then *μ*_1_ > 0 and *v*_1_ = *Av*_1_ + *μ*_1_ *φ*_1*h*_⩾*μ*_1_ *φ*_1*h*_. Let

(53) $$\begin{array}{c} \mu^{*}=\sup\left\{\mu >0:v_{1}⩾\mu \varphi_{1h}\right\}. \end{array}$$

Then μ*⩾*μ*_1_ > 0 and *v*_1_⩾μ*φ_1*h*_. Since *T*_*h*_ is a positive linear operator, we have

(54) $$\begin{array}{c} \lambda_{1h}T_{h}v_{1}⩾\mu^{*}\lambda_{1h}T_{h}\varphi_{1h}=\mu^{*}\varphi_{1h}. \end{array}$$

Hence, by (52) we have

(55) v1=Av1+μ1φ1h⩾λ1hThv1+μ1φ1h⩾μ∗φ1h+μ1φ1h,

which contradicts the definition of μ*. Thus according to the property of omitting a direction for fixed point index, we have *i*(*A*, *Q*∩*Ω*_*r*_, *Q*) = 0.

(vii) From (39), there exist *η* > 0 and *ε*_0_ ∈ (0,1) such that

(56) $$\begin{array}{c} q\left(v\right)⩾\lambda_{1h}\left(1+\varepsilon_{0}\right)v\;\forall v\in \left[\eta ,+\infty \right). \end{array}$$

Since *q* is bounded on [0, *η*], there is a constant *C*_2_ > 0 such that

(57) $$\begin{array}{c} q\left(v\right)⩾\lambda_{1h}\left(1+\varepsilon_{0}\right)v-C_{2}\;\forall v\in \left[0,\eta \right]. \end{array}$$

Thus *q*(*v*)⩾*λ*_1*h*_(1 + *ε*_0_)*v* − *C*_2_ for all *v* ∈ [0, +*∞*). Hence, by (38) we have

(58) $$\begin{array}{c} g\left(t,v\right)⩾\lambda_{1h}\left(1+\varepsilon_{0}\right)h\left(t\right)v-C_{2}h\left(t\right) \end{array}$$

for all (*t*, *v*)∈(0,1)×[0, +*∞*). Let *C*_3_ = *C*_2_∫_0_^1^ *h*(*t*)*φ*_1*h*_(*t*)(∫_0_^1^ *k*(*t*, *s*)*h*(*s*)*ds*)*dt*; then *C*_3_ > 0 is a finite constant. Take

(59) $$\begin{array}{c} R>C_{3}{\left(\varepsilon_{0}\min\left\{a,1-b\right\}\overset{b}{\underset{a}{\int }}h\left(t\right)\varphi_{1h}\left(t\right)dt\right)}^{-1}. \end{array}$$

Suppose that there exist *v*_1_ ∈ *Q*∩∂*Ω*_*R*_ and *μ*_1_⩾0 such that *v*_1_ = *Av*_1_ + *μ*_1_ *φ*_1*h*_; then

(60) Jh(v1) =J(Av1)+μ1J(φ1h) ⩾J(Av1) ⩾∫01h(t)φ1h(t)(λ1h(1+ε0)∫01k(t,s)h(s)v1(s)ds−C2Th(1))dt =λ1h(1+ε0)Jh(Thv1)−C3 =(1+ε0)Jh(v1)−C3.

Hence,

(61) $$\begin{array}{c} J_{h}\left(v_{1}\right)⩽C_{3}\varepsilon_{0}^{-1}. \end{array}$$

On the other hand,

(62) Jh(v1)=∫01h(t)φ1hv1(t)dt⩾∫abh(t)φ1hv1(t)dt⩾Rmin⁡{a,1−b}∫abh(t)φ1h(t)dt.

By the maximum principle, *φ*_1*h*_(*t*) > 0 for all *t* ∈ (0,1). By *h*(*t*)≢0 for *t* ∈ [*a*, *b*], we have ∫_*a*_^*b*^ *h*(*t*)*φ*_1*h*_(*t*)*dt* > 0. Thus from (62) and (61), we have

(63) R⩽(min⁡{a,1−b}∫abh(t)φ1h(t)dt)−1Jh(v1)⩽C3(ε0min⁡{a,1−b}∫abh(t)φ1h(t)dt)−1.

This is a contradiction. So, by the property of omitting a direction for fixed point index, we have *i*(*A*, *Q*∩*Ω*_*R*_, *Q*) = 0. The proof is completed.

Now, we are ready to state our main results of this section.

**Theorem 7**

Assume that (H_1_), (H_2_), (H_3_), and (H_6_) hold; then the singular BVP (7) has at least two positive solutions.

**Proof**

According to Lemma 6, we can choose sufficiently small positive number *r* and sufficiently large positive number *R* satisfying 0 < *r* < *l* < *R*, *i*(*A*, *P*∩*Ω*_*r*_, *P*) = 1, and *i*(*A*, *P*∩*Ω*_*R*_,  *P*) = 1. From *i*(*A*, *P*∩*Ω*_*l*_, *P*) = 0 and the additivity property of the fix point index, we obtain

(64) $$\begin{array}{c} i\left(A,P\cap \left(\Omega_{l}\setminus {\bar{\Omega }}_{r}\right),P\right)=0-1=-1,\\ i\left(A,P\cap \left(\Omega_{R}\setminus {\bar{\Omega }}_{l}\right),P\right)=1-0=1. \end{array}$$

Hence *A* has at least two fixed points, one in $\Omega_{l}\setminus {\bar{\Omega }}_{r}$ and another in $\Omega_{R}\setminus {\bar{\Omega }}_{l}$. That is, the singular BVP (7) has at least two positive solutions. The proof is completed.

**Theorem 8**

If (H_1_) and one of the following conditions are satisfied, then the singular BVP (7) has at least one positive solution.

- (H_2_) and (H_5_) hold;
- (H_2_) and (H_6_) hold;
- (H_2_) and (H_8_) hold;
- (H_3_) and (H_4_) hold;
- (H_3_) and (H_6_) hold;
- (H_3_) and (H_7_) hold.

**Proof**

By the properties of the fixed point index, we only need to choose suitable positive numbers *r* and *R*. This completes the proof.

**Remark**

The following conditions are little stronger than (H_1_), (H_2_), and (H_3_), respectively. And they are somewhat easy to verify. In fact, (H_1_*) is a key condition in [19].

(H_1_*) *g* ∈ *C*((0,1) × ℝ^+^, ℝ^+^) and there exist *h* ∈ *H* and *ψ* ∈ *C*(ℝ^+^, ℝ^+^) such that *g*(*t*, *v*) ⩽ *h*(*t*)*ψ*(*v*) for all (*t*, *v*)∈(0,1) × ℝ^+^;
(H_2_*) limsup⁡_*v*→0+_ *ψ*(*v*)/*v* < *λ*_1*h*_;
(H_3_*) limsup⁡_*v*→*∞*_ *ψ*(*v*)/*v* < *λ*_1*h*_.

If (H_1_*) holds, then, for any *M* > 0, let *h*_*M*_(*t*) = *h*(*t*)max⁡_*v*∈[0,*M*]_ *ψ*(*v*); then we have *g*(*t*, *v*) ⩽ *h*(*t*)*ψ*(*v*) ⩽ *h*_*M*_(*t*) for all (*t*, *v*)∈(0,1)×[0, *M*] and *h*_*M*_ ∈ *H*. Consequently, (H_1_) holds. From *g*(*t*, *v*)/*h*(*t*)*v* ⩽ *h*(*t*)*ψ*(*v*)/*h*(*t*)*v* = *ψ*(*v*)/*v*, it is easy to see that if (H_2_*) or (H_3_*) holds, then (H_2_) or (H_3_) holds, respectively.

**Remark 10**

In [11], Stańczy established a one-solution theorem for the singular problem (7). In addition to (H_1_), the key conditions imposed on the nonlinear term *g* are the following ones:

(A2) there exists a set *A* ⊂ (0,1) of positive measures such that
  (65) $$\begin{array}{c} \underset{v\to +\infty }{\lim}\frac{g\left(t,v\right)}{v}=\infty \;\text{uniformly}\;\;\text{w}.\text{r}.\text{t}.\;t\in A; \end{array}$$
(A3) there exists a function *h* ∈ *H* such that
  (66) $$\begin{array}{c} \underset{v\to 0+}{\lim}\frac{g\left(t,v\right)}{h\left(t\right)v}=0\;\text{uniformly}\;\;\text{w}.\text{r}.\text{t}.\;\;t\in \left(0,1\right). \end{array}$$

It is easy to see that (A2) is equivalent to the following condition:

(A2′) there exists a set [*a*, *b*]⊂(0,1) such that
  (67) $$\begin{array}{c} \underset{v\to +\infty }{\lim}\;\underset{t\in [a,b]}{\min}\frac{g\left(t,v\right)}{v}=\infty . \end{array}$$

In a recent paper [4], Han and Wang improved the results in [11] by substituting the following conditions for the above ones:

 (HW5) liminf⁡_*v*→*∞*_min⁡_*t*∈[*a*,*b*]_⁡*g*(*t*, *v*)/*v* > *M*_1_;
 (HW2) there exists a function *h* ∈ *H* such that
  (68) $$\begin{array}{c} \underset{v\to 0+}{\mathrm{limsup}}\frac{g\left(t,v\right)}{h\left(t\right)v}<m_{h}\;\text{uniformly}\;\;\text{w}.\text{r}.\text{t}.\;t\in \left(0,1\right), \end{array}$$

where *m*_*h*_ = (max⁡_*t*∈[0,1]_∫_0_^1^ *k*(*t*, *s*)*h*(*s*)*ds*)^−1^ for *h* ∈ *H*.

Additionally, they obtained a twin-solution theorem for the singular BVP (7) by using (HW2), (H_6_), and

 (HW3) there exists a function *h* ∈ *H* such that
  (69) $$\begin{array}{c} \underset{v\to +\infty }{\mathrm{limsup}}\frac{g\left(t,v\right)}{h\left(t\right)v}<m_{h}\;\text{uniformly}\;\;\text{w}.\text{r}.\text{t}.\;t\in \left(0,1\right). \end{array}$$

Since *m*_*h*_ ⩽ *λ*_1*h*_, (H_2_) is an improvement of (HW2). In fact, without loss of generality, suppose *φ*_1*h*_, the positive eigenfunction corresponding to *λ*_1*h*_, satisfies ||*φ*_1*h*_|| = *φ*_1*h*_(*t*_0_) = 1; then

(70) 1=φ1h(t0)=λ1h∫01k(t0,s)h(s)φ1h(s)ds⩽λ1h∫01k(t0,s)h(s)ds⩽λ1hmax⁡t∈[0,1]∫01k(t,s)h(s)ds.

So *m*_*h*_ ⩽ *λ*_1*h*_ and then (HW2) implies (H_2_). Therefore, Theorems 7 and 8 are essential improvements of Theorems 3.1, 3.2 in [4] and Theorem 2.2 in [11]. Furthermore, we give the new conditions (H_7_) and (H_8_). These improvements allow us to deal with more singular problems.

**Remark 11**

For the following singular BVP with general boundary conditions,

(71) $$\begin{array}{c} -v^{\prime \prime }\left(t\right)=g\left(t,v\left(t\right)\right)\;\text{for}\;t\in \left(0,1\right),\\ \alpha v\left(0\right)-\beta v^{\prime }\left(0\right)=0,\;\;\;\gamma v\left(1\right)+\delta v^{\prime }\left(1\right)=0, \end{array}$$

where *α*, *β*, *γ*, and *δ*⩾0 and *ρ* = *γβ* + *αγ* + *αδ* > 0, our results still hold. In fact, the Green function

(72) $$\begin{array}{c} G\left(t,s\right)=\{\begin{array}{cc} \frac{1}{\rho }\left(\gamma +\delta -\gamma t\right)\left(\beta +\alpha s\right), & 0⩽s⩽t⩽1,\\ \frac{1}{\rho }\left(\gamma +\delta -\gamma s\right)\left(\beta +\alpha t\right), & 0⩽t⩽s⩽1, \end{array} \end{array}$$

has analogous properties as *k*(*t*, *s*) is defined in (10) (see [4, Remark 3.3]). Hence, Theorems 7, 8 can be generalized to the singular problem (71) without any essential difficulty. See [4, 19, 20] for details.

For the following singular two-point BVP whose variables of nonlinear term are separated, the hypotheses and results will be more concise:

(73) $$\begin{array}{c} -v^{\prime \prime }=h\left(t\right)g\left(v\right),\;t\in \left(0,1\right),\\ v\left(0\right)=v\left(1\right)=0. \end{array}$$

Since *h* is a fixed function, *T*_*h*_, *λ*_1*h*_, and *φ*_1*h*_ are confirmed exclusively. So we skip the subscript *h* in the following. Corresponding  to (H_1_)–(H_8_), we formulate the conditions for singular BVP (73):

(H_1_′) *g* ∈ *C*(ℝ^+^, ℝ^+^) and *h* ∈ *H*;
(H_2_′) limsup⁡_*v*→0+_ *g*(*v*)/*v* < *λ*_1_;
(H_3_′) limsup⁡_*v*→+*∞*_ *g*(*v*)/*v* < *λ*_1_;
(H_4_′) min⁡_*t*∈[*a*,*b*]_⁡*h*(*t*) > 0 and liminf⁡_*v*→0+_ *g*(*v*)/*v* > *M*_1_(min⁡_*t*∈[*a*,*b*]_ *h*(*t*))^−1^;
(H_5_′) min⁡_*t*∈[*a*,*b*]_⁡*h*(*t*) > 0 and liminf⁡_*v*→+*∞*_ *g*(*v*)/*v* > *M*_1_(min⁡_*t*∈[*a*,*b*]_⁡*h*(*t*))^−1^;
(H_6_′) there exists a number *l* > 0 such that
  (74) $$\begin{array}{c} h\left(t\right)g\left(v\right)>\lambda l\;\text{for}\;\left(t,v\right)\in \left[a,b\right]\times \left[\min\left\{a,1-b\right\}l,l\right]; \end{array}$$
(H_7_′) liminf⁡_*v*→0+_ *g*(*v*)/*v* > *λ*_1_;
(H_8_′) liminf⁡_*v*→+*∞*_ *g*(*v*)/*v* > *λ*_1_.

**Remark**

Take *t*_1_ ∈ [*a*, *b*] with *φ*_1_(*t*_1_) = min⁡_*t*∈[*a*,*b*]_ *φ*_1_(*t*). Then

(75) φ1(t1)=λ1∫01k(t1,s)h(s)φ1(s)ds⩾λ1∫abk(t1,s)h(s)φ1(s)ds⩾λ1φ1(t1)∫abk(t1,s)h(s)ds⩾λ1φ1(t1)min⁡t∈[a,b]∫abk(t,s)h(s)ds⩾λ1φ1(t1)min⁡t∈[a,b]h(t)min⁡t∈[a,b]∫abk(t,s)ds.

Notice that *φ*_1_(*t*_1_), min⁡_*t*∈[*a*,*b*]_ *h*(*t*), and min⁡_*t*∈[*a*,*b*]_∫_*a*_^*b*^ *k*(*t*, *s*)*ds* > 0; we have

(76) $$\begin{array}{c} \lambda_{1}⩽M_{1}{\left(\underset{t\in \left[a,b\right]}{\min}h(t)\right)}^{-1}. \end{array}$$

So (H_4_′) implies (H_7_′) and (H_5_′) implies (H_8_′).

**Remark 13**

Observe that the condition

(77) $$\begin{array}{c} h\left(t\right)≢0\;\text{for}\;t\in \left[a,b\right] \end{array}$$

is not contained in (H_8_′). In fact, the cone *Q* will be replaced by the cone *P*_0_ which is defined in (15) as we consider one solution by (H_8_′). See the proof of Theorem 15 in the following.

**Theorem 14**

Assume that (H_1_′), (H_2_′), (H_3_′), and (H_6_′) hold; then the singular BVP (73) has at least two positive solutions.

**Proof**

This theorem is a direct corollary of Theorem 7.

**Theorem 15**

If (H_1_′)  and one of the following conditions are satisfied, then the singular BVP (7) has at least one positive solution.

- (H_2_′) and (H_5_′) hold;
- (H_2_′) and (H_6_′) hold;
- (H_2_′) and (H_8_′) hold;
- (H_3_′) and (H_4_′) hold;
- (H_3_′) and (H_6_′) hold;
- (H_3_′) and (H_7_′) hold.

**Proof**

(i), (ii), and (iv)–(vi) are direct corollaries of Theorem 7. Next, we prove (iii). In contrast to (H_8_), (H_8_′) does not contain the condition that *h*≢0 for *t* ∈ [*a*, *b*].

(iii) Since  *T*(*P*) ⊂ *P*_0_ is completely continuous, we know that *A*(*P*) ⊂ *P*_0_ is also completely continuous. Similar to item (i) of Lemma 6, by (H_2_′), it is not difficult to prove *i*(*A*, *P*_0_∩*Ω*_*r*_, *P*_0_) = 1 for sufficiently small positive number *r*. By (H_8_′), there exist *η* > 0 and *ε*_0_ ∈ (0,1) such that

(78) $$\begin{array}{c} g\left(v\right)⩾\lambda_{1}\left(1+\varepsilon_{0}\right)v\;\;\forall v\in \left[\eta ,+\infty \right). \end{array}$$

Since *g* is bounded on [0, *η*], there is a constant *C*_4_ > 0 such that *g*(*v*)⩾*λ*_1_(1 + *ε*_0_)*v* − *C*_4_ for all *v* ∈ [0, *η*]. Thus

(79) $$\begin{array}{c} g\left(v\right)⩾\lambda_{1}\left(1+\varepsilon_{0}\right)v-C_{4}\;\;\forall v\in \left[0,+\infty \right). \end{array}$$

Let *C*_5_ = *C*_4_∫_0_^1^ *h*(*t*)*φ*_1_(*t*)(∫_0_^1^ *k*(*t*, *s*)*h*(*s*)*ds*)*dt*; then *C*_5_ > 0 is a finite constant. Take

(80) $$\begin{array}{c} R>\max\left\{r,C_{5}\lambda_{1}\delta_{1}^{-1}\varepsilon_{0}^{-1}\right\}. \end{array}$$

Suppose that there exist *v*_1_ ∈ *P*_0_∩∂*Ω*_*R*_ and *μ*_1_⩾0 such that *v*_1_ = *Av*_1_ + *μ*_1_ *φ*_1_; then from (79) we have

(81) J(v1)=J(Av1)+μ1J(φ1)⩾J(Av1)⩾∫01h(t)φ1(t)(λ1(1+ε0)∫01k(t,s)h(s)v1(s)ds−C4T(1))dt=λ1(1+ε0)J(Tv1)−C5=(1+ε0)J(v1)−C5.

Hence,

(82) $$\begin{array}{c} J\left(v_{1}\right)⩽C_{5}\varepsilon_{0}^{-1}. \end{array}$$

On the other hand, since *v*_1_ ∈ *P*_0_∩∂*Ω*_*R*_, we have

(83) $$\begin{array}{c} J\left(v_{1}\right)⩾\lambda_{1}^{-1}\delta_{1}||v_{1}||=\lambda_{1}^{-1}\delta_{1}R. \end{array}$$

Thus from (82) and (83), we have

(84) $$\begin{array}{c} R⩽\lambda_{1}\delta_{1}^{-1}J\left(v_{1}\right)⩽C_{5}\lambda_{1}\delta_{1}^{-1}\varepsilon_{0}^{-1}. \end{array}$$

This contradicts with (80). By the property of omitting a direction for fixed point index, we have *i*(*A*, *P*_0_∩*Ω*_*R*_, *P*_0_) = 0. So $i(A,P_{0}\cap (\Omega_{R}\setminus {\bar{\Omega }}_{r}))=0-1=-1$. Thus *A* has a fixed point in $\Omega_{R}\setminus {\bar{\Omega }}_{r}$; that is, the singular BVP (73) has at least one positive solution. The proof is completed.

**Remark 16**

Lan and Webb have studied BVP (73) in [21]. Their key conditions are

- *h* ∈ *L* ^1^(0,1), *h*⩾0 a.e. on [0,1] and *g* ∈ *C*(ℝ^+^, ℝ^+^);
- One of the following conditions holds:
  (h1) limsup⁡_*v*→0+_ *g*(*v*)/*v* < *α* and *β* < liminf⁡_*v*→+*∞*_ *g*(*v*)/*v*;
  (h2) limsup⁡_*v*→+*∞*_ *g*(*v*)/*v* < *α* and *β* < liminf⁡_*v*→0+_ *g*(*v*)/*v*, where *α* = *m*_*h*_ and *β* = (min⁡_*t*∈[*a*,*b*]_∫_*a*_^*b*^ *k*(*t*, *s*)*h*(*s*)*ds*)^−1^.

Under the above condition (i), we still can prove Lemma 2 (see Theorem 2.1 in [21]). By (70) and (75), we have *α* ⩽ *λ*_1*h*_ ⩽ *β*. Therefore, the items (iii) and (vi) of Theorem 15 extend the main results in [21] essentially. Furthermore, our conditions (H_2_′), (H_3_′), (H_7_′), and (H_8_′) cannot be improved anymore.

At the end of this section, we present three simple examples to which our theorems can be applied, respectively. We choose [*a*, *b*] = [1/4,3/4].

**Example**

Let

(85) g(t,v)={1t(1−t)(cvt+1),v∈[0,18l],1t(1−t) ×[(88−c)(v−18l)t+18clt+1],v∈[18l,14l],1t(1−t)(11lt+1),v∈[14l,l],1t(1−t)[c(v−l)+11lt+1],v∈[l,+∞],

where *c* > 0 is a constant. Obviously, *g*(*t*, *v*) ⩽ *h*(*t*)*ψ*(*v*) for all (*t*, *v*)∈(0,1) × ℝ^+^, where *h*(*t*) = *t* ^−1^(1 − *t*)^−1^ and

(86) $$\begin{array}{c} \psi \left(v\right)=\{\begin{array}{cc} cv+2, & v\in \left[0,\frac{1}{8}l\right],\\ \left(88-c\right)\left(v-\frac{1}{8}l\right)+\frac{1}{8}cl+2, & v\in \left[\frac{1}{8}l,\frac{1}{4}l\right],\\ 11l+2, & v\in \left[\frac{1}{4}l,l\right],\\ c\left(v-l\right)+11l+2, & v\in \left[l,+\infty \right]. \end{array} \end{array}$$

Since *λ* = 32/3 < 11, if lim⁡_*v*→0+_ *ψ*(*v*)/*v* = *c* < *λ*_1*h*_ and lim⁡_*v*→+*∞*_ *ψ*(*v*)/*v* = *c* < *λ*_1*h*_, then *g* satisfies all the conditions of Theorem 7; thus, we infer that the singular BVP (7) has at least two positive solutions. Furthermore, if

(87) mh=(max⁡t∈[0,1]∫01k(t,s)h(s)ds)−1=(ln⁡2)−1≈1.443<c<λ1h,

then Theorem 3.1 in [4] is invalid for this example.

**Example 18**

Let

(88) g(t,v)=11−tcv+12t(1−t)v1/2+11−t=1t(1−t)(tcv+12v1/2+t)⩽1t(1−t)(cv+12v1/2+1)≜h(t)ψ(v),

where 0 < *c* < *λ*_1*h*_. Since lim⁡_*v*→0+_ *ψ*(*v*)/*v* = +*∞* > *λ*_1*h*_ and lim⁡_*v*→+*∞*_ *ψ*(*v*)/*v* = *c* < *λ*_1*h*_, the item (iv) of Theorem 8 implies that singular BVP (7) has at least one positive solution.

**Example 19**

Let

(89) $$\begin{array}{c} g\left(t,v\right)=h\left(t\right)\psi \left(v\right)=t^{p-1}{\left(1-t\right)}^{q-1}\overset{n}{\underset{i=1}{\sum }}c_{i}v^{i}, \end{array}$$

where *p*, *q* ∈ (0,1), *a*_*i*_ > 0 for *i* = 1,2,…, *n*, and *a*_1_ < *λ*_1*h*_. Since lim⁡_*v*→0+_ *ψ*(*v*)/*v* = *a*_1_ < *λ*_1*h*_ and lim⁡_*v*→+*∞*_ *ψ*(*v*)/*v* = +*∞* > *λ*_1*h*_, the item (iii) of Theorem 15 ensures that the singular BVP (73) has at least one positive solution.

## 3. Positive Radial Solutions of Elliptic Boundary Value Problems

Define a set

(90) K={p∈C(1,∞,ℝ+):p≢0, ∫1∞s(1−s2−n)p(s)ds<+∞}.

Denote *c* = (1 − *a*)^1/(2−*n*)^ and *d* = (1 − *b*)^1/(2−*n*)^. For *p* ∈ *K*, let

(91) $$\begin{array}{c} h\left(t\right)=\frac{1}{{\left(n-2\right)}^{2}}s^{2n-2}p\left({\left(1-t\right)}^{1/(2-n)}\right). \end{array}$$

Then we have *h* ∈ *H*. As in (13) and Lemma 2, *h* confirms an operator *T*_*h*_ and its first eigenvalue *λ*_1*h*_. To emphasize their relation with *p*, we use the notations *h*_*p*_, *λ*_1*h*_*p*__, and *φ*_1*h*_*p*__.

According to (8), we formulate the following conditions which correspond to those in Section 2.

(C_1_) *f* ∈ *C*((1, *∞*) × ℝ^+^, ℝ^+^) and for any *M* > 0 there exists a function *p*_*M*_ ∈ *K* such that
  (92) $$\begin{array}{c} f\left(s,u\right)⩽p_{M}\left(s\right)\;\forall \left(s,u\right)\in \left(1,\infty \right)\times \left[0,M\right]; \end{array}$$
(C_2_) there exists a function *p* ∈ *K* such that
  (93) $$\begin{array}{c} \underset{u\to 0+}{\mathrm{limsup}}\frac{f\left(s,u\right)}{p\left(s\right)u}<\lambda_{1h_{p}}\;\text{uniformly}\;\text{w}.\text{r}.\text{t}.\;s\in \left(1,\infty \right); \end{array}$$
(C_3_) there exists a function *p* ∈ *K* such that
  (94) $$\begin{array}{c} \underset{u\to +\infty }{\mathrm{limsup}}\frac{f\left(s,u\right)}{p\left(s\right)u}<\lambda_{1h_{p}}\;\text{uniformly}\;\text{w}.\text{r}.\text{t}.\;s\in \left(1,\infty \right); \end{array}$$
(C_4_) liminf⁡_*u*→0+_min⁡_*s*∈[*c*,*d*]_⁡*f*(*s*, *u*)/*u* > *c* ^2−2*n*^(*n* − 2)^2^ *M*_1_;
(C_5_) liminf⁡_*u*→+*∞*_min⁡_*s*∈[*c*,*d*]_⁡*f*(*s*, *u*)/*u* > *c* ^2−2*n*^(*n* − 2)^2^ *M*_1_;
(C_6_) there exists a number *l* > 0 such that
  (95) f(s,u)>(n−2)2λlfor  (s,u)∈[c,d]×[min⁡{a,1−b}l,l];
(C_7_) there exists a function *p* ∈ *K* such that
  (96) $$\begin{array}{c} \underset{u\to 0+}{\mathrm{liminf}}\frac{f\left(s,u\right)}{p\left(s\right)u}>\lambda_{1h_{p}}\;\text{uniformly}\;\text{w}.\text{r}.\text{t}.\;s\in \left(1,\infty \right); \end{array}$$
(C_8_) there exist *p* ∈ *K* with *p*(*s*)≢0 for *s* ∈ (*c*, *d*) and *q* ∈ *C*(ℝ^+^, ℝ^+^) such that
  (97) f(s,u)⩾p(s)q(u) ∀(s,u)∈(1,+∞)×ℝ+,liminf⁡u→+∞q(u)u>λ1hp.

Now we are ready to state our main results for the elliptic BVP (1).

**Theorem**

If (C_1_), (C_2_), (C_3_), and (C_6_) are satisfied, then the elliptic BVP (1) has at least two positive radial solutions.

**Theorem 21**

If (C_1_) and one of the following conditions are satisfied, then the elliptic BVP (1) has at least one positive radial solution:

- (C_2_) and (C_5_) hold;
- (C_2_) and (C_6_) hold;
- (C_2_) and (C_8_) hold;
- (C_3_) and (C_4_) hold;
- (C_3_) and (C_6_) hold;
- (C_3_) and (C_7_) hold.

**Remark 22**

According to Remark 10, we know

(98) mp=(n−2)(max⁡τ∈[0,1]∫1∞k(τ,1−s2−n)sn−1p(s)ds)−1<λ1hp for  p∈K.

Therefore, Theorems 20 and 21 are essential improvements of Theorems 4.1 and 4.2 in [4], respectively.

**Remark 23**

For the following BVP whose nonlinear terms are variable-separated, it is easy to give more concise theorems as Theorems 14 and 15; we skip them here:

(99) $$\begin{array}{c} -\Delta u=p\left(\left|x\right|\right)f\left(u\right)\;\text{for}\;\;\left|x\right|>1,\;x\in {\mathbb{R}}^{n},\;n⩾3,\\ u=0\;\text{for}\;\;\left|x\right|=1,\\ u⟶0\;\text{as}\;\;\left|x\right|⟶+\infty , \end{array}$$

where *p* ∈ *C*((1, *∞*), ℝ^+^) and *f* ∈ *C*(ℝ^+^, ℝ^+^).

**Remark 24**

One can see that, through variables substitution, it is easy to give some examples like in Section 3, so we skip it.
